# High-quality genome assembly of *Pseudocercospora ulei* the main threat to natural rubber trees

**DOI:** 10.1590/1678-4685-GMB-2021-0051

**Published:** 2022-01-05

**Authors:** Sandra González-Sayer, Ursula Oggenfuss, Ibonne García, Fabio Aristizabal, Daniel Croll, Diego M. Riaño-Pachon

**Affiliations:** 1 Universidade de São Paulo, Centro de Energia Nuclear na Agricultura, Laboratório de Biologia Computacional, Evolutiva e de Sistemas, Piracicaba, SP, Brazil.; 2 Universidad Nacional de Colombia, Instituto de Biotecnología, Laboratorio de Caracterización Molecular, Bogotá D.C., Colombia.; 3 Neuchâtel University, Laboratory of Evolutionary Genetics, Neuchâtel, Switzerland.

**Keywords:** Natural rubber, South American Leaf Blight, PacBio, Nanopore, transposable elements

## Abstract

*Pseudocercospora ulei* is the causal agent of South American Leaf Blight (SALB), the main disease affecting *Hevea brasiliensis* rubber tree, a native species to the Amazon. Rubber tree is a major crop in South American countries and SALB disease control strategies would benefit from the availability of genomic resources for the fungal pathogen. Here, we assembled and annotated the *P. ulei* genome. Shotgun sequencing was performed using second and third generation sequencing technologies. We present the first *P. ulei* high-quality genome assembly, the largest among Mycosphaerellaceae, with 93.8 Mbp, comprising 215 scaffolds, an N50 of 2.8 Mbp and a BUSCO gene completeness of 97.5%. We identified 12,745 protein-coding gene models in the *P. ulei* genome with 756 genes encoding secreted proteins and 113 genes encoding effector candidates. Most of the genome (80%) is composed of repetitive elements dominated by retrotransposons of the *Gypsy* superfamily. *P. ulei* has the largest genome size among Mycosphaerellaceae, with the highest TE content. In conclusion, we have established essential genomic resources for a wide range of studies on *P. ulei* and related species.

Natural rubber, a polymer composed of isoprene monomers, is the primary raw material for a broad range of industrial products (Yamashita and Takahashi, 2020). *Hevea brasiliensis* (Muell. Arg., Euphorbiaceae), a species native to the Amazon rainforest, is the main source of natural rubber. Natural rubber production in South America is seriously restricted by the ascomycetous fungus *Pseudocercospora ulei* (Henn.) Hora Júnior and Mizubuti, the causal agent of the South American Leaf Blight (SALB) disease (Lieberei, 2007). SALB causes defoliation, impacting photosynthetic capability, reducing rubber production of up to 75%, and possible death of the susceptible trees (Chee, 1976). Despite the agronomic importance of *P. ulei*, knowledge about the molecular mechanisms governing its biological functions, i.e., pathogenicity, is missing. This lack of knowledge about *P. ulei* is largely due to difficulties growing the fungus outside of its host (Chee, 1978), as *P. ulei* is thought to be biotroph (Lieberei, 2006). *P. ulei* has several reproduction stages (one teleomorph and two anamorphs), however it is only possible to propagate *in vitro* the conidial form of the fungus (Guyot and Doaré, 2010). Based on recent molecular phylogenetic analyses, *P. ulei* was recently placed in the Mycosphaerellaceae family of Dothideomycetes in a clade including *P. fijiensis*, the main pathogen of the banana crop (Hora *et al*., 2014). Other species in the same family are associated with a wide range of diseases on economically relevant hosts. This work reports a high-quality genome assembly for *P. ulei* highlighting the large, transposable element-rich genome making it the biggest assembled genome in the Mycosphaerellaceae family.

The *P. ulei* GCL012 strain was obtained from the *H. brasiliensis* GT1 accession established at the Agrosavia clonal garden in Villavicencio - la Libertad, Colombia. The source for DNA and RNA extraction were stromata of the isolated *P. ulei* GCL012. Conidia were originally collected from a single plant lesion (Figure 1a-b) and propagated on M3 solid medium at 25 ºC in the dark for 45 days. The stromata obtained were propagated in M4 solid medium (Figure 1c). Growth and sporulation were stimulated by light exposition for 90 minutes/day (Lieberei, 2007). High molecular weight DNA was extracted using a CTAB DNA extraction protocol (Stirling, 2003) and sequenced by a shotgun hybrid sequencing approach, combining second and third generation sequencing technologies. A total of 2,019,638 raw reads with an N50 of 5,103 bp were generated on a Pacific BioSciences Sequel instrument by Novogene (USA). In addition, 3,606,462 raw reads with an N50 of 4,506 bp were produced *in house* on a MinION portable sequencer using a single 1D Genomic DNA library (Oxford Nanopore Technologies - ONT; Ligation library prep kit - SQK-LSK109). Additionally, 18,492,732 paired-end reads (2x250 bp) were generated on an Illumina HiSeq 2500 instrument, by Novogene (USA), using the TruSeq library preparation kit. Short reads were trimmed with Trimmomatic (see programs versions, and references in Table S1) using Phred score-33, leading-3, trailing-6, sliding-window-4:20, milen:80 parameters, and cleaned from possible contaminants using bbduk.

**Figure 1 -:**
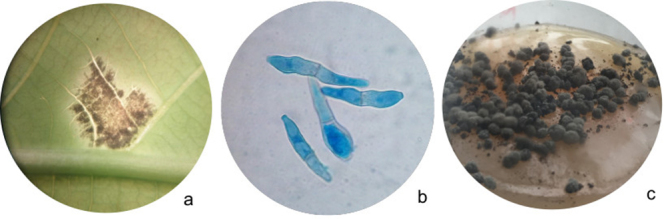
*P. ulei in vitro* culture: isolation and stroma propagation. a. A single plant lesion, magnification 1x. b. *P. ulei* conidias stained with lactophenol blue solution, magnification 100x. c. Growth of stroma in an Erlenmeyer 125 ml with sporulation artificial media (M4) at 14 days. Photos from the Molecular Characterization Laboratory, Biotechnology Institute, National University of Colombia - Natural Rubber Research. Photos taken by Sandra Milena Gonzalez-Sayer (a and c) and Maria Elizabeth Mendez Tibambre (b). Photo a), taken with Advanced optical stereoscopy, serial:0709011, Photo b), taken with Olympus Microscopy CH30 model, serial: T39D15816. Photo c), taken with an iPhone 6s camera.

Non-hybrid, hybrid and hierarchical assembly methods were assessed exploiting *P. ulei* second and third generation sequencing reads (Table S2). The best assembly was generated with Canu using the PacBio dataset with a corrected error rate of 0.045 at normal coarse sensitivity level and polished twice with Pilon using the Illumina dataset. Genome scaffolding was performed identifying *kmer* pairs between ONT corrected reads and the assembled contigs using LINKs. Completeness of the genome assembly was assessed by Benchmarking Universal Single-Copy Orthologous (BUSCO) software with the Ascomycete dataset from OrthoDB v.9. The assembled *P. ulei* genome has 93.8 Mb and was fragmented into 231 contigs, where the largest was 11.2 Mbp, (N50 = 2.35 Mbp), GC% = 50.27% (Table 1). BUSCO identified 1,282 complete orthologous genes (97.5%) in the assembly. From these, 1,273 were single-copy, and only 9 (0.7%) were duplicated, confirming that this genome is haploid (Table 1). The 231 *P. ulei* genome contigs were processed into 215 scaffolds; 17 scaffolding events increased the N50 value to 2.82 Mbp (Table 1). The *P. ulei* whole-genome assembly was deposited in GenBank with the accession number JACWNB000000000 under BioProject PRJNA661960.

**Table 1 -:** Assembly quality metrics of the scaffolded *P. ulei* genome.

Quast Metrics	*P.ulei*_Canu_scaffolded	
# Contigs	215	
Longest Contig	11,240,615	
Total length	93,730,151	
N50	2,827,303	
L50	8	
contigs (>= 10000 bp)	179	
Number of scaffolds >50kb	70	
Predicted coding-regions genes	12,745	
GC (%)	50.27	
BUSCOs Category	Nº	%
Complete BUSCOs	1,282	97.5
Complete single copy	1,273	96.8
Complete single duplicated	9	0.7
Fragmented	13	1.0
Missing	20	1.5
Total BUSCOs searched	1,315	

We performed the taxonomic placement of *P. ulei* GCL012 among the *Pseudocercospora* genus by phylogenetic and phylogenomic approaches. The phylogenetic analysis was based on four genomic loci: actin, partial transcription elongation Factor 1-α (EF-1α) and ITS 1 and ITS2, including 278 *Pseudocercospora* species (Table S3). For that, gene sequences were identified via BLAST searchers with seed sequences (Table S4), and further refined with EMBOSS. Final sequences were aligned with MAFFT using ‘auto’ alignment option for the Actin and EF-1α loci and ‘mafft-qinsi’ for the ITS 1 and 2 loci. Poorly aligned regions were identified and removed with TrimAl using the -automated1 mode and Jalview Software. We carried out a partitioned phylogenetic inference with IQ-Tree, using a partition matrix generated with FASconCAT-G. The evolutionary models for each locus used for the phylogenetic inference were Actin:TIM2e+G4, EF-1α:TN+F+G4,TPM2+F+R3, and ITS1-ITS2:TPM2+F+G4. This phylogenetic analysis showed many deeply branching lineages of low support (Figure S1). Recent discovery of new *Pseudocercospora* species in sampling expeditions from tropical and subtropical environments (Bakhshi *et al*., 2014; Silva *et al*., 2016) and taxonomy studies (Quaedvlieg *et al*., 2012; Crous *et al*., 2013; Bakhshi *et al*., 2014; Nakashima *et al*., 2016; Silva *et al*., 2016), had revealed that the *Pseudocercospora* genus is highly diverse and has not been fully explored yet. Nevertheless, we could identify four deeply branching clades with good support, Clades A to D (Figure S1). *P. ulei* was not assigned to either of these four clades, but the topology suggests a relationship to Clades B and C. The closest relative of *P. ulei* is *P. camelliicola*, a pathogen of camellia cultivars.

We then carried out a phylogenetic inference using 1,315 orthologous gene sequences extracted from the genome sequences of seven *Pseudocercospora* species, and 37 species outside *Pseudocercospora* genus (Table S5). Amino acid sequences from orthologous genes shared between the 44 assessed species were independently aligned with MAFFT and trimmed with trimAL. A maximum likelihood phylogeny was estimated with IQ-Tree using 1,000 bootstrap replicates. A supertreee was inferred using Astral. We confirmed previously published findings indicating that *P. fijiensis*, *P. eumusae*, *P. musae*, *P. cruenta* and *P. pini_densiflora* are grouping in a single clade (Clade D in the 4-marker phylogeny Figure S1) distinct from *P. ulei* and *P. macadamia* (Figure 2). All these analyses clearly place *P. ulei* in a clade which has not been well explored from a genomics point of view.

**Figure 2 -:**
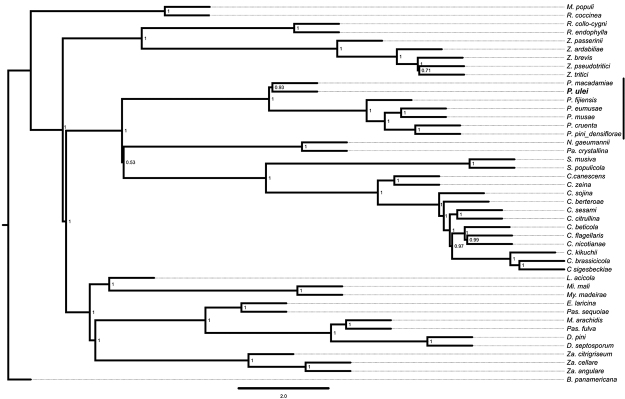
Phylogenetic inference of members of Mycosphaerellaceae for which genome sequences are available (see Table S5 for the genome accession number for each species). Phylogenetic tree inferred with a supertree approach using Astral, using the protein sequences of 1,315 orthologous genes identified by BUSCO. Individual gene trees were inferred with IQ-Tree. *P. ulei* appears in bold face. Member of the genus *Pseudocercospora* are identified by a vertical black line.

Genome annotation was performed with Braker2 training predictors with identified BUSCO genes, protein sequences from *Pseudocercospora* spp., and RNAseq reads from *P. fijiensis* (SRP075820). To assist the prediction of gene models, we generated *P. ulei* RNAseq data from two developmental stages of *in vitro* culture: 13th day of growth and 18th day of growth using the NucleoSpin® RNA Plant and Fungi MACHEREY-NAGEL kit (Düren, Germany). A total of 341,630,429 PE-150bp strand-specific raw RNAseq reads were produced in a Illuminas’ NovaSeq 6000 instrument (San Diego, CA). RNAseq reads were mapped against the *P. ulei* genome using HISAT2 and converted into hints using bam2-hints. Functional annotation of the predicted proteins was performed with InterProScan. Secreted proteins were predicted with SignalP, Phobious, and Wolfsorf. The sub-cellular localization was predicted using ApoplastP. Small secreted proteins (SSP), Carbohydrate Active Enzymes (CAZymes), and secondary metabolite gene clusters were predicted with effectorP, dbCAN2 and AntiSMASH, respectively. The *P. ulei* lifestyle was inferred with CATAStrophy.

The *P. ulei* genome encodes a total of 12,745 protein-coding genes, with a mean length of 2,075 bp and a gene complete BUSCO content of 91.3%. Approximately 5.7% (n=756) of the predicted proteins are secreted and 113 classified as putative effectors including 54 presenting potential activity in the host apoplast. 1.6% percent (n=216) of the proteins are classified as CAZymes. The number of CAZymes in this genome is among the lowest in a set of 69 mycelial ascomycetes (Hane et al., 2020), and in the seven *Pseudocercospora* spp. sequenced genomes. The low proportion of CAZyme content has been described as an evolutionary trait of hemibiotrophic and biotrophic lifestyles within the Capnodiales (Haridas *et al*., 2020). CATAStrophy is a genome-informed trophic classifier for filamentous fungi based on the content of CAZyme genes (Hane *et al*., 2020). From the low number of predicted CAZyme genes in *P. ulei*’s genome, many of which are involved in the degradation of simple sugars, CATAStrophy predicts that *P. ulei* is a monomerthroph (Hane *et al*., 2020). This is also supported by the low number of gene clusters predicted for secondary metabolites. 

Transposable element families were identified based on a consensus library including the *Pseudocercospora* genomes generated with RepeatModeler and annotated with RepeatMasker. Remarkably, The *P. ulei* genome shows an exceptionally high repetitive content (80.6%). 79% of the genome are TEs, dominated by the superfamily Gypsy (46.1%). Genomes sizes differ among Dothideomycetes with a strong correlation to transposable element content (Haridas *et al*., 2020). *Pseudocercospora* species show similar correlations, with the Sigatoka complex of banana pathogens carrying the biggest genomes. *P. fijiensis* and *P. musae* have 52.7 and 50.1% of TE content, respectively (Chang *et al*., 2016). The smallest genomes (*P. macadamia* and *P. pini*) have fewer transposable elements. *P. ulei* has the biggest known genome so far reported in the Mycosphaerellaceae family (size rank: 26.5 Mbp - 74.1 Mbp - Table S5) and with the highest TE content (Figure S2). Although the reasons underlying this burst TEs in *P. ulei*, or its effects, are not yet clear, changes in the number of TE insertion have been associated to the responses to biotic and abiotic stressors (Fouché *et al*., 2019). And even though TE proliferation was often believed to mainly have deleterious effects (Horváth *et al*., 2017), TE insertions can also have beneficial effects, particularly as a source of regulatory sequences (Lisch, 2013; Chuong *et al*., 2017). A detailed study of *P. ulei’*s TEs will be published elsewhere.

*P. ulei* has the most complex genome so far reported among Mycosphaerellaceae, it has the largest genome with the highest content of TEs (79%). Our genome assembly using second and third generation sequencing technologies posits this genome as the second best in terms of contiguity (L75) among the genomes with more than 45Mbp in the family (Figure S3). 

## Supplementary material

The following online material is available for this article:

Table S1 - Listing of all software packages used in this work, with version numbers and references.

Table S2 - Quality metrics from the assembly strategies assessed to reconstruct the *P. ulei* genome.

Table S3 - Accession numbers for the sequences of the four phylogenetic markers (actin, partial transcription elongation Factor 1-α (EF-1α) and ITS 1 and ITS2) in 278 *Pseudocercospora* species, used for the phylogenetic reconstruction shown in Figure S1.

Table S4 - Accession number of seed sequences used to identify the four phylogenetic makers (Figure S1) through BLAST searchers in the target *Pseudocercospora* species.

Table S5 - Accession numbers for the genome sequences of species within Mycosphaerellaceae used for phylogenomic analyses.

Figure S1 -
*P. ulei* phylogenetic replacement based on genomic loci (actin, partial transcription elongation Factor 1-α (EF-1α), ITS 1 and ITS2) assessed in the *Pseudocercospora* genus.

Figure S2 - Relative content of transposable elements (TEs) in the genome sequences of *Pseudocercospora* species.

Figure S3 - Relationship between genome assembly contiguity, genome assembly size and number of contigs/scaffolds for all the genomes of species of Mycosphaerellaceae.
