# Supplementary material for: High-quality genome assembly of Pseudocercospora ulei the main threat to natural rubber trees
Source: Genet Mol Biol. 2022 Jan 5;45(1):e50510051. doi: 10.1590/1678-4685-GMB-2021-0051 (PMC8762716; doi:10.1590/1678-4685-GMB-2021-0051)
Supplement: Table S1 - [file 1415-4757-GMB-45-1-e20210051-s1.pdf]

Genetics and Molecular Biology
https://doi.org/10.1590/1678-4685-GMB-2021-0051

Supplementary Material to "High-quality genome assembly of *Pseudocercospora ulei* the main threat to natural rubber trees"

Table S1 - Listing of all software packages used in this work, with version numbers and references.

Program	Version	Reference
AntiSMASH	v.5	Blin K, Medema M, Kazempour D, Fischbach M, Breitling R, Takano E, et al (2013) antiSMASH 2.0 a versatile platform for genome mining of secondary metabolite producers. Nucleic Acids Res. 41(1), Pages 204-212.
Trimmomatic	v.0.38	Bolger A, Lohse M and Usadel B (2014) Trimmomatic: a flexible trimmer for illumina sequence data. Bioinformatics, Volume 30, Issue 15, 1 August 2014, Pages 2114–2120.
Bbmap/bbduck	v. 35.85	Bushnell B (2014) BBMap: A fast, accurate, splice-aware-aligner. LBNL Department of energy joint genome institute, 2800 Mitchell Drive, Walnut Creek, USA.
TrimAl	v.1.4rev22	Capella S, Silla J and Gabaldón T (2009) TrimAl: a tool for automated alignment trimming in large-scale phylogenetic analyses. Bioinformatics. 25(15): Pages 1972- 3.
RepeatMasker	v.open-4.0.9	Graovac M and Chen N (2009) Using RepeatMasker to identify repetitive elements in genomic sequences. Curr Protoc. Bioinformatics 25, 4.10.1–4.10.14.
CATAStrophy	v.0.1.0	Hane J, Paxman J, Jones D, Oliver R and de Wit Pierre (2020) "CATAStrophy," a genome-informed trophic classification of filamentous plant pathogens – how many different types of filamentous plant pathogens are there?. Front Microbiol(10): 3088: Pages 1-12.
Braker2	v.2.1	Hoff K, Lange S, Lomsadze A, Borodovsky M and Stanke M (2015) BRAKER1: unsupervised RNA-Seq-based genome annotation with GeneMark-ET and AUGUSTUS. Bioinformatics 32: Pages 767–769.
Wolfsorf	v. 0.2	Horton P, Park K, Obayashi T, Fujita N, Harada H, Adams-Collier C and Nakai K (2007) WoLF PSORT: protein localization predictor. Nucleic Acids Res. Jul; 35(Web Server issue): Pages 585–587.
InterProScan	v.5.36-75	Jones P, Binns D, Chang H, Fraser M, Li W, McAnulla C, MacWillian H, Maslen J, Mitchel A and Nuka G (2014) InterProScan 5: genome-scale protein function classification. Bioinformatics. 30(9): Pages 1236–40.

Program	Version	Reference
Phobious	November 20/2020 – online version	Käll L. Krogh A and Sonnhammer E (2007) Advantages of combined transmembrane topology and signal peptide prediction—the Phobius web server. Nucleic Acids Res. Jul; 35(Web Server issue): Pages 429-32.
MAFFT	v.7.407	Katoh K, Kuma K, Toh H, Miyata T (2005) MAFFT version 5: Improvement in accuracy of multiple sequences alignment. Nucleic Acids Res 33: Pages 511-518.
HISAT2	v.2.0.5	Kim D, Langmead B and Salzberg S (2015) HISAT: a fast spliced aligner with low memory requirements. Nat Methods 12: Pages 357–360.
Canu	v.1.8	Koren S, Brian P, Walenz Berlin K, Miller J, Bergman N and Phillip A (2017) Canu: scalable and accurate long-read assembly via adaptive k-mer weighting and repeat separation. May; 27(5): Pages 722–736.
FASconCAT-G	v.1.04	Kück P and Longo G (2014) FASconCAT-G: extensive functions for multiple sequence alignment preparations concerning phylogenetic studies. Front Zool 11, Page 81.
IQTree	v.1.6.9	Lam-Tung N, Heiko A, Schmidt Arndt von H and Bui M (2015) IQ-TREE: A fast and effective stochastic algorithm for estimating maximum-likelihood phylogenies, Mol Biol Evol, Volume 32, Issue 1, Pages 268–274.
SignalP	v.5.0b	Nielsen H (2017) Predicting secretory proteins with SignalP. In: Kihara D, editor. protein function prediction: methods and protocols. New York, NY: Springer New York; Pages 59–73.
EMBOSS	v.6.0.0	Rice P. Longden I and Bleasby A (2000) EMBOSS: The European molecular biology open software suite. Trends Genet. 16(6): Pages 276-277.
BUSCO	v.3.0.2	Simão F, Waterhouse R, Evgenia P, Kriventseva E, Zdobnov M (2015) BUSCO: assessing genome assembly and annotation completeness with single-copy orthologs, Bioinformatics, Volume 31, Issue 19, Pages 3210–3212.
RepeatModeler	v.1.0.11	Smit A, Hubley R and Green P (2015) Repeat Masker Open-4.0 . Available from: http://repeatmasker.org
ApoplastP	v.1.0	Sperschneider J, Dodds PN, Singh KB and Taylor J (2018) ApoplastP: prediction of effectors and plant proteins in the apoplast using machine learning. New Phytol, 217(4): Pages 1764-1778.
EffectorP	v.2.0	Sperschneider J, Gardiner D, Dodds P, Tini F, Covarelli L, Singh K, et al. (2015). EffectorP: predicting fungal effector proteins from secretomes using machine learning. New Phytol. 210(2): Pages 743–61.
Augustus/bam2hints	v. 3.3	Stanke M, Keller O, Gunduz I, Hayes A, Waack S and Morgenstern B (2006) AUGUSTUS: *ab initio* prediction of alternative transcripts. Nucleic Acids Res; (Web Server issue): Pages 4–9.
Pilon	v.1.23	Walker B, Abeel T, Shea T, Priest M, Abouelliel A, Sakthikumar S, Cuomo C, Zeng Q, Wortman J, Young S and Earl A (2014) Pilon: an integrated tool for comprehensive microbial variant detection and genome assembly improvement. PLoS One 9:e112963
LINKs	v.1.8.6	Warren R, Yang C, Vandervalk B, Behsaz B, Lagman A, Jones S and Birol I (2015) LINKS: Scalable, alignment-free scaffolding of draft genomes with long reads. Aug 4; 4:35.
Jalview	v.2.11.1.3	Waterhouse A, Procter J, Martin D, Clamp M and Barton G (2009) Jalview Version 2-a multiple sequence alignment editor and analysis workbench. Bioinformatics 25: Pages 1189-1191.

**Program**	**Version**	**Reference**
dbcan2	Last update 09/08/19	Yin Y, Mao X, Yang J, Chen X, Mao F and Xu Y (2012) dbCAN: a web resource for automated carbohydrate-active enzyme annotation. Nucleic Acids Res. 40(Web Server issue): Pages 445–451.
Astral	v.5.7.3	Zhang C, Maryam R, Erfan S and Siavash M (2018) "ASTRAL-III: Polynomial Time Species Tree Reconstruction from Partially Resolved Gene Trees." BMC Bioinformatics 19 (S6): Page 153.
