# Supplementary material for: High-quality genome assembly of Pseudocercospora ulei the main threat to natural rubber trees
Source: Genet Mol Biol. 2022 Jan 5;45(1):e50510051. doi: 10.1590/1678-4685-GMB-2021-0051 (PMC8762716; doi:10.1590/1678-4685-GMB-2021-0051)
Supplement: Table S2 - [file 1415-4757-GMB-45-1-e20210051-s2.pdf]

*Genetics and Molecular Biology*
https://doi.org/10.1590/1678-4685-GMB-2021-0051

# Supplementary Material to "High-quality genome assembly of *Pseudocercospora ulei* the main threat to natural rubber trees"

**Table S2 -** Quality metrics from the assembly strategies assessed to reconstruct the *P. ulei* genome.

| Assembly | Short reads assemblies (Only Illumina reads) | | Hybrid assemblies (Illumina, Pacbio and ONT reads) | | Long non-hybrid assemblies (Only PacBio reads) | | | Long non-hybrid assemblies (Only ONT reads) | Long hybrid (PacBio and ONT reads) |
|---|---|---|---|---|---|---|---|---|---|
| **Assembly** | **Short_SOAP** | **Short_Masurca** | **Hybrid_Masurca** | **SPAdes** | **PacB_Wtdbg2** | **PacB_Falcon** | **PacB_Canu** | **Nano_canu** | **Nano_Pab_canu** |
| **STATISTICS WITHOUT REFERENCE** | | | | | | | | | |
| # Contigs | 36,541 | 13,776 | 1,193 | 3,804 | 1,311 | 573 | 231 | 167 | 336 |
| Longest Contig | 54,712 | 97,624 | 91,1021 | 1,095,545 | 617,313 | 2,072,718 | 11,256,605 | 4,202,079 | 5,800,913 |
| Total length | 50,382,058 | 84,979,727 | 85,638,836 | 92,612,415 | 92,666,995 | 83,710,889 | 93,812,866 | 91,182,491 | 95,849,438 |
| N50 | 1,623 | 11,182 | 110,987 | 147,130 | 143,999 | 280,044 | 2,355,240 | 1,465,888 | 2,123,084 |
| GC (%) | 51.4 | 50.16 | 50.58 | 50.19 | 50.23 | 50.71 | 50.27 | 50.19 | 50.27 |

**BUSCOs RESULTS**

| **Category** | Short_SOAP **Nº** | Short_SOAP **%** | Short_Masurca **Nº** | Short_Masurca **%** | Hybrid_Masurca **Nº** | Hybrid_Masurca **%** | SPAdes **Nº** | SPAdes **%** | PacB_Wtdbg2 **Nº** | PacB_Wtdbg2 **%** | PacB_Falcon **Nº** | PacB_Falcon **%** | PacB_Canu **Nº** | PacB_Canu **%** | Nano_canu **Nº** | Nano_canu **%** | Nano_Pab_canu **Nº** | Nano_Pab_canu **%** |
|---|---|---|---|---|---|---|---|---|---|---|---|---|---|---|---|---|---|---|
| Complete BUSCOs | 1,191 | 90.5 | 1,226 | 93.3 | 1,228 | 94.4 | 1,281 | 97.4 | 1,205 | 91.7 | 932 | 70.9 | 1,282 | 97.5 | 1,090 | 82.9 | 1,260 | 95.8 |
| Complete single copy | 1,188 | 90.3 | 1,225 | 93.2 | 1,208 | 91.9 | 1,280 | 97.3 | 1,204 | 91.6 | 932 | 70.9 | 1,273 | 96.8 | 1,089 | 82.8 | 1,244 | 94.6 |
| Complete single duplicated | 3 | 0.2 | 1 | 0.1 | 20 | 1.5 | 1 | 0.1 | 1 | 0.1 | 0 | 0.0 | 9 | 0.7 | 1 | 0.1 | 16 | 1.2 |
| Fragmented | 94 | 7.1 | 58 | 4.4 | 17 | 1.3 | 13 | 1.0 | 51 | 3.9 | 158 | 12.0 | 13 | 1.0 | 100 | 7.6 | 23 | 1.7 |
| Missing | 30 | 2.4 | 31 | 2.3 | 70 | 5.3 | 21 | 1.6 | 59 | 4.4 | 225 | 17.1 | 20 | 1.5 | 125 | 9.5 | 32 | 2.5 |
| Total BUSCO groups searched | 1,315 | | | | | | | | | | | | | | | | | |
