# Supplementary material for: High-quality genome assembly of Pseudocercospora ulei the main threat to natural rubber trees
Source: Genet Mol Biol. 2022 Jan 5;45(1):e50510051. doi: 10.1590/1678-4685-GMB-2021-0051 (PMC8762716; doi:10.1590/1678-4685-GMB-2021-0051)
Supplement: Table S3 - [file 1415-4757-GMB-45-1-e20210051-s3.pdf]

Genetics and Molecular Biology
https://doi.org/10.1590/1678-4685-GMB-2021-0051

Supplementary Material to "High-quality genome assembly of *Pseudocercospora ulei* the main threat to natural rubber trees"

Table S3 - Accession numbers for the sequences of the four phylogenetic markers (actin, partial transcription elongation Factor 1-α (EF-1α) and ITS 1 and ITS2) in 278 *Pseudocercospora* species, used for the phylogenetic reconstruction shown in Figure S1.

SPECIES	ITS	EF-1α	ACT
Pseudocercospora abelmoschi	GU269647	GU384365	GU320355
Pseudocercospora acericola	GU269650	GU384368	GU320358
Pseudocercospora ampelopsis	GU269830	GU384542	GU320534
Pseudocercospora angolensis	GU269836	GU384548	JQ325010
Pseudocercospora araliae	GU269652	GU384370	GU320360
Pseudocercospora arcuata	GU269850.1	JQ325006.1	GU320554.1
Pseudocercospora arecacearum	GU269655	GU384373	GU320363
Pseudocercospora assamensis	GU269656	GU384374	GU320364
Pseudocercospora atromarginalis	GU269658	GU384376	GU320365
Pseudocercospora balsaminae	GU269660	GU384379	GU320367
Pseudocercospora basiramifera	GU269661	GU384492	GU320368
Pseudocercospora basitruncata	GU269662	DQ211675	DQ147622
Pseudocercospora callicarpae	GU269663	GU384380	GU320369
Pseudocercospora catalpigena	GU269690	GU384406	GU320395
Pseudocercospora catappae	GU269669	GU384386	GU320375
Pseudocercospora cercidicola	GU269671	GU384388	GU320377
Pseudocercospora cercidis-chinensis	GU269670	GU384387	GU320376
Pseudocercospora cf. Kaki	GU269728	GU384441	GU320430
Pseudocercospora cheg	GU269673	GU384390	GU320379
Pseudocercospora chionanthi-retusi	GU269674	GU384391	GU320380
Pseudocercospora chrysanthemicola	GU269675	GU384392	GU320381
Pseudocercospora cladosporioides	GU269678	GU384395	GU320383
Pseudocercospora colombiensis	AY752149	DQ211660	DQ147639
Pseudocercospora contraria	GU269677	GU384394	GU320385
Pseudocercospora coprosmae	GU269680	GU384397	GU320386
Pseudocercospora cordiana	GU269681	GU384398	GU320387
Pseudocercospora coriariae	GU269682	GU384399	GU320388
Pseudocercospora cornicola	GU269683	GU384400	GU320389
Pseudocercospora corylopsidis	GU269721	GU384437	GU320425
Pseudocercospora cotoneastri	GU269685	GU384402	GU320391
Pseudocercospora crispans	GU269807	GU384518	GU320510
Pseudocercospora crocea	GU269792	GU384502	GU320493
Pseudocercospora crousii	GU269686	GU384403	GU320392
Pseudocercospora cruenta	GU269688	GU384404	JQ325012
Pseudocercospora cydoniae	GU269691	GU384407	GU320396

SPECIES	ITS	EF-1α	ACT
Pseudocercospora cymbidiicola	GU269692	GU384408	GU320397
Pseudocercospora davidiicola	GU269693	GU384409	GU320398
Pseudocercospora dendrobii	GU269696	GU384412	GU320401
Pseudocercospora destructiva	GU269694	GU384410	GU320399
Pseudocercospora dianellae	GU269695	GU384411	GU320400
Pseudocercospora dodonaeae	GU269697	GU384413	JQ325013
Pseudocercospora dovyalidis	GU269800	GU384513	GU320503
Pseudocercospora elaeocarpi	GU269701	GU384417	GU320405
Pseudocercospora eucalyptorum	GU269659	DQ211678	DQ147614
Pseudocercospora eupatoriella	GU269704	GU384420	GU320408
Pseudocercospora eustomatis	GU269705	GU384421	GU320409
Pseudocercospora exosporioides	GU269707	GU384423	GU320411
Pseudocercospora fijiensis (CIRAD 86)	KB446561	KB446555	KB446556
Pseudocercospora flavomarginata	GU269799	GU384512	DQ166513
Pseudocercospora fori	GU269806	GU384517	DQ147618
Pseudocercospora fraxinites	GU269672	GU384389	GU320378
Pseudocercospora fukuokaensis	GU269713	GU384429	GU320417
Pseudocercospora fuligena	GU269711	GU384427	GU320415
Pseudocercospora glauca	GU269715	GU384431	GU320419
Pseudocercospora gracilis	DQ267582	DQ211666	DQ147616
Pseudocercospora griseola f. griseola	GU269717	GU384433	GU320421
Pseudocercospora guianensis	GU269719	GU384435	GU320423
Pseudocercospora haiweiensis	GU269803	GU384514	GU320506
Pseudocercospora hakeae	GU269784	GU384495	JQ325017
Pseudocercospora humuli	GU269725	GU384439	GU320428
Pseudocercospora humulicola	GU269724	JQ324996	JQ325018
Pseudocercospora indonesiana	GU269735	GU384448	GU320437
Pseudocercospora ixorae	GU269726	GU384440	GU320429
Pseudocercospora jussiaeae	JQ324977	JQ324998	JQ325020
Pseudocercospora kakiicola	GU269729	GU384442	GU320431
Pseudocercospora kiggelariae	GU269730	GU384443	GU320432
Pseudocercospora latens	GU269732	GU384445	GU320434
Pseudocercospora leucadendri	GU269842	GU384555	GU320545
Pseudocercospora libertiae	GU269733	GU384446	GU320435
Pseudocercospora lilacis	GU269737	GU384449	GU320439
Pseudocercospora longispora	GU269734	GU384447	GU320436
Pseudocercospora lonicericola	GU269736	JQ324999	GU320438
Pseudocercospora luzardii	GU269738	GU384450	GU320440
Pseudocercospora lyoniae	GU269739	GU384451	GU320441
Pseudocercospora lythracearum	GU269740	GU384452	GU320442
Pseudocercospora lythri	GU269742	GU384454	GU320444
Pseudocercospora macrospora	GU269745	GU384457	GU320447
Pseudocercospora mali	GU269744	GU384456	GU320446
Pseudocercospora marginalis	GU269794	GU384504	GU320495
Pseudocercospora metrosideri	GU269746	GU384458	GU320448
Pseudocercospora musae	GU269747	GU384459	GU320449
Pseudocercospora myrticola	GU269749	GU384460	GU320451
Pseudocercospora nandinae	GU269750	GU384461	GU320452
Pseudocercospora natalensis	GU269780	JQ325000	DQ147620
Pseudocercospora nephrolepidis	GU269751	GU384462	GU320453

SPECIES	ITS	EF-1α	ACT
Pseudocercospora nogalesii	GU269752	GU384463	GU320454
Pseudocercospora norchiensis	GU269772	GU384484	GU320455.1
Pseudocercospora ocimi-basilici	GU269754	GU384465	GU320456
Pseudocercospora oenotherae	GU269856	GU384567	GU320559
Pseudocercospora palleobrunnea	GQ303288	GU384509	GU320500
Pseudocercospora pallida	GU269758	GU384469	GU320459
Pseudocercospora pancratii	GU269759	GU384470	GU320460
Pseudocercospora paraguayensis	DQ267602	DQ211680	DQ147606
Pseudocercospora pini-densiflorae	GU269760	GU384471	GU320461
Pseudocercospora plecthranthi	GU269791	GU384501	GU320492
Pseudocercospora pouzolziae	GU269761	GU384472	GU320462
Pseudocercospora profusa	GU269787	GU384497	GU320488
Pseudocercospora proteae	GU269808	GU384519	GU320511
Pseudocercospora prunicula	GU269676	GU384393	GU320382
Pseudocercospora pseudostigmina-platani	GU269857	GU384568	GU320560
Pseudocercospora puderi	GU269764	GU384476	GU320467
Pseudocercospora punctata	GU269765	GU384477	GU320468
Pseudocercospora purpurea	GU269783	GU384494	GU320486
Pseudocercospora pyracanthae	GU269767	GU384479	GU320470
Pseudocercospora ranjita	GU269790	GU384500	GU320491
Pseudocercospora ravenalicola	GU269810	GU384521	GU320513
Pseudocercospora rhabdothamni	GU269768	GU384480	GU320471
Pseudocercospora rhamnellae	GU269795	GU384505	GU320496
Pseudocercospora rhapisicola	GU269770	GU384482	GU320473
Pseudocercospora rhoina	GU269771	GU384483	GU320474
Pseudocercospora robusta	MH862873	DQ211683	DQ147617
Pseudocercospora rubi	GU269773	GU384485	GU320476
Pseudocercospora rumohrae	GU269774	GU384486	GU320477
Pseudocercospora sambucigena	GU269788	GU384498	GU320489
Pseudocercospora securinegae	GU269776	GU384487	GU320479
Pseudocercospora sordida	GU269777	GU384488	GU320480
Pseudocercospora stahlii	GU269813	GU384525	GU320515
Pseudocercospora stephanandrae	GU269814	GU384526	GU320516
Pseudocercospora subsessilis	GU269815	GU384527	GU320517
Pseudocercospora subtorulosa	GU269816	GU384528	GU320518
Pseudocercospora subulata	DQ303090	JQ325004	GU320519
Pseudocercospora tereticornis	JQ324982	GU384377	JQ325025
Pseudocercospora theae	GU269821	GU384534	GU320524
Pseudocercospora tibouchinigena	GU269822	GU384535	GU320525
Pseudocercospora timorensis	GU269823	GU384536	GU320526
Pseudocercospora variicolor	GU269826	GU384538	GU320530
Pseudocercospora viburnigena	GU269809	GU384520	GU320512
Pseudocercospora viticicola	GU269828	GU384540	GU320532
Pseudocercospora vitis	GU269829	GU384541	GU320533
Pseudocercospora weigelae	GU269831	GU384543	GU320535
Pseudocercospora xanthocercidis	JQ324983	JQ325005	JQ325026
Pseudocercospora xanthoxyli	GU269832	GU384544	GU320536
Pseudocercospora zelkovae	GU269833	GU384545	JQ325027
Pseudocercospora aeschynomenicola	KT290146	KT290200	KT313501
Pseudocercospora bixae	KT290153	KT290207	KT313508
Pseudocercospora boehmeriigena	KT290152	KT290206	KT313507

SPECIES	ITS	EF-1α	ACT
Pseudocercospora chamaecristae	KT290147	KT290201	KT313502
Pseudocercospora diplusodonii	KT290135	KT290189	KT313490
Pseudocercospora emmotunicola	KT290136	KT290190	KT313491
Pseudocercospora euphorbiacearum	KT313500	KT290199	KT313500
Pseudocercospora exilis	KT290139	KT290193	KT313494
Pseudocercospora manihotii	KT290144	KT290198	KT313499
Pseudocercospora perae	KT290132	KT290186	KT313487
Pseudocercospora Planaltinensis	KT290137	KT290191	KT313492
Pseudocercospora plumeriifolii	KT290138	KT290192	KT313493
Pseudocercospora plunkettii	KT290151	KT290205	KT313506
Pseudocercospora pothomorphes	KT290131	KT290185	KT313486
Pseudocercospora richardsoniicola	KT290154	KT290208	KT313509
Pseudocercospora Rigidae	KT290134	KT290188	KT313489
Pseudocercospora sennae-multijugae	KT290142	KT290196	KT313497
Pseudocercospora solani-pseudocapsicicola	KT290148	KT290202	KT313503
Pseudocercospora stizolobii	KT290143	KT290197	KT313498
Pseudocercospora struthanthi	KT290141	KT290195	KT313496
Pseudocercospora tecomicola	KT290156	KT290209	KT313511
Pseudocercospora wulffiae	KT290150	KT290204	KT313505
Pseudocercospora xylopiae	KT290133	KT290187	KT313488
Pseudocercospora eumusae	LFZN01000734.1	LFZN01000037.1	LFZN01000053.1
Pseudocercospora ulei	SCF 35	SCF 1	SCF 23
Pseudocercospora macadamia	WRNY01000139	KU878503	WRNY01000012
Pseudocercospora Piperis	JX875062	JX896123	-
Pseudocercospora prunicola	GU269676	GU384393	GU320382
Pseudocercospora pyracanthigena	GU269766	GU384478	GU320469
Pseudocercospora Sawadae	GU269775	-	GU320478
Pseudocercospora sp CBS 110998	GU269778	GU384489	GU320481
Pseudocercospora trinidadensis	KT290157	KT290210	-
Pseudocercospora udagawana	GU269824	GU384537	GU320527
Pseudocercospora vassobiae	KT290155	-	KT313510
Pseudocercospora ershadii	KM452865	KM452887	KM452842
Pseudocercospora fuliginosa	LC515776	LC515789	LC515779
Pseudocercospora kobayashiana	LC511998	LC515780	LC512004
Pseudocercospora diospyriphila	LC512003	LC515790	GU512009
Pseudocercospora paederiae	GU269757	GU384468	-
Pseudocercospora rhododendri-indici	GU269722	-	GU320426
Pseudocercospora snelliana	GU269731	GU384444	GU320433
Pseudocercospora thailandica	-	GU384533	GU320523
Pseudocercospora chibaensis	KX462584	KX462670	KX462551
Pseudocercospora cyathicola	JF951139	KX462673	KX462554
Pseudocercospora daphniphylli	KX462587	KX462674	KX462555
Pseudocercospora elaeocarpicola	KX462588	KX462675	KX462556
Pseudocercospora Eriobotryae	KX462589	KX462676	KX462557
Pseudocercospora eriobotryicola	KX462589	KX462677	KX462558
Pseudocercospora eupatorii-formosani	KX462591	KX462678	KX462559
Pseudocercospora hachijokibushii	KX462593	KX462680	KX462561
Pseudocercospora hiratsukana	KX462594	KX462681	KX462562
Pseudocercospora houttuyniae	KX462595	KX462682	KX462563

SPECIES	ITS	EF-1α	ACT
Pseudocercospora imazekii	KX462607	KX462693	KX462574
Pseudocercospora izuohshimense	KX462597	KX462684	KX462565
Pseudocercospora kadsurae	KX462598	KX462685	KX462566
Pseudocercospora madagascariensis	GQ852767	KF253265	KF253625
Pseudocercospora naitoi	KX462599	KX462686	KX462567
Pseudocercospora nephrolepidicola	HQ599590	KX462688	KX462569
Pseudocercospora neriicola	KJ869165	KJ869240	KJ869231
Pseudocercospora photiniae	KX462604	KX462690	KX462571
Pseudocercospora punicae	KX462606	KX462692	KX462573
Pseudocercospora tinea	KX462608	KX462696	GU320499
Pseudocercospora violamaculans	KX462610	KX462698	KX462579
Pseudocercospora xenosyzygiicola	KX462611	KX462699	KX462580
Pseudocercospora pyricola	KY048161.1	KY048164.1	KX462580.1
Pseudocercospora airliensis	KM055429.1	KM055436.1	-
Pseudocercospora jagerae	KM055431.1	KM055438.1	-
Pseudocercospora proiphydis	KM055430.1	KM055437.1	-
Pseudocercospora iwakiensis	KX462607.1	KX462693.1	KX462574.1
Pseudocercospora piricola	KY048161.1	KY048164.1	KY048162.1
Pseudocercospora aristoteliae	MK432806.1	-	-
Pseudocercospora amelanchieris	KX462583.1	KX462669.1	KX462550.1
Pseudocercospora athyrii	KJ201932.1	-	-
Pseudocercospora beijingensis	MH255813.1	MH255819.1	MH392526.1
Pseudocercospora bischofiae	KC677896.1	-	-
Pseudocercospora brackenicola	KT037524.1	KT037484.1	-
Pseudocercospora breonadiae	MH107913.1	MH108026.1	MH107985.1
Pseudocercospora buteae	KC677909.1	-	-
Pseudocercospora camelliicola	KJ201933.1	-	-
Pseudocercospora carbonacea	KC677897.1	-	-
Pseudocercospora casuarinae	HQ599603.1	-	-
Pseudocercospora chiangmaiensis	MH863288.1	KF903177.1	KF903544.1
Pseudocercospora christellae	KC677898.1	-	-
Pseudocercospora circumscissa	KX853044.1	KX853061.1	-
Pseudocercospora cladrastidis	AB694923.1	-	-
Pseudocercospora clematidis	DQ303072.1	JX901677.1	JX902133.1
Pseudocercospora clerodendri-hastati	LC146754.1	-	-
Pseudocercospora cyatheae	KJ201935.1	-	-
Pseudocercospora depazeoides	LT160035.1	-	-
Pseudocercospora dingleyae	KX287299.1	-	-
Pseudocercospora duabangae	KC677899.1	-	-
Pseudocercospora ebulicola	KJ201936.1	-	-
Pseudocercospora elaeocarpi	GU269701.1	GU384417.1	GU320405.1
Pseudocercospora elaeodendri	KC172073.1	-	-
Pseudocercospora euonymi	MH255812.1	MH255818.1	MH392525.1
Pseudocercospora fatouae	DQ303076.1	-	-
Pseudocercospora fici	KJ412456.1	-	-
Pseudocercospora ficisepticae	MW063148.1	-	-
Pseudocercospora ginkgoana	JX134048.1	-	-
Pseudocercospora gmelinae	KC677901.1	-	-
Pseudocercospora hamiltoniani	MN737835.1	-	-
Pseudocercospora heteropyxidicola	MN562151.1	-	-

SPECIES	ITS	EF-1α	ACT
Pseudocercospora hibbertiae-asperae	AF488743.1	-	-
Pseudocercospora holarrhenae	KC677902.1	-	-
Pseudocercospora jahnii	KM393283.1	-	-
Pseudocercospora kamalii	JF824126.1	-	-
Pseudocercospora leandrae-fragilis	KY574288.1	-	-
Pseudocercospora lindericola	KX853047.1	-	-
Pseudocercospora ygodiicola	KT037526.1	-	-
Pseudocercospora macarangae	KC677905.1	-	-
Pseudocercospora maetaengensis	MN648323.1	-	-
Pseudocercospora malloticola	KC677906.1	-	-
Pseudocercospora mapelanensis	KM203118.1	-	-
Pseudocercospora mazandaranensis	KM452855.1	KM452877.1	KM452832.1
Pseudocercospora microlepiae	KR348740.1	-	-
Pseudocercospora micromeli	KC677910.1	-	-
Pseudocercospora mombin	KC677907.1	-	-
Pseudocercospora nelumbonicola	KY304493.1	-	-
Pseudocercospora nodosa	MF951367.1	-	-
Pseudocercospora nymphaeacea	KT074354.1	KT074355.1	KT074353.1
Pseudocercospora oenotherae	MK863390.1	GU384466.1	GU320559.1
Pseudocercospora opuntiae	KF975410.1	-	-
Pseudocercospora paranaensis	KT037523.1	KT037483.1	KT037604.1
Pseudocercospora parapseudarthriae	KJ869151.1	-	-
Pseudocercospora pittospori	MK210511.1	-	-
Pseudocercospora platylobii	AY260089.1	-	-
Pseudocercospora protearum	AY251107.2	-	-
Pseudocercospora pseudomyrticola	MK876405.1	MK876499.1	MK876461.1
Pseudocercospora pteridicola	KR348738.1	-	-
Pseudocercospora pteridophytophila	KJ201938.1	-	-
Pseudocercospora puerariicola	MG601511.1	-	-
Pseudocercospora rhododendrigena	LC146756.1	-	-
Pseudocercospora rosae	MG828952.1	-	-
Pseudocercospora schizolobii	JQ676195.1	JX901695.1	KF253628.1
Pseudocercospora serpocaulonicola	KT037525.1	KT037485.1	KT037607.1
Pseudocercospora sphaerulinae	JX901791.1	KF903215.1	-
Pseudocercospora ubsynnematosa	KF686808.1	-	-
Pseudocercospora syzygiicola	AF309600.1	-	-
Pseudocercospora tabernaemontanae	KC677911.1	-	-
Pseudocercospora utamarindi	KP744461.1	-	-
Pseudocercospora thelypteridis	KT037521.1	-	-
Pseudocercospora tibouchinicola	KF686809.1	-	-
Pseudocercospora tibouchinigena	GU269822.1	-	-
Pseudocercospora trichogena	KT037520.1	KT037480.1	KT037601.1
Pseudocercospora wedeliae	KJ201940.1	-	-
