# Supplementary material for: High-quality genome assembly of Pseudocercospora ulei the main threat to natural rubber trees
Source: Genet Mol Biol. 2022 Jan 5;45(1):e50510051. doi: 10.1590/1678-4685-GMB-2021-0051 (PMC8762716; doi:10.1590/1678-4685-GMB-2021-0051)
Supplement: Table S4 - [file 1415-4757-GMB-45-1-e20210051-s4.pdf]

Genetics and Molecular Biology
https://doi.org/10.1590/1678-4685-GMB-2021-0051

Supplementary Material to "High-quality genome assembly of *Pseudocercospora ulei* the main threat to natural rubber trees"

Table S4 - Accession number of seed sequences used to identify the four phylogenetic makers (Figure S1) through BLAST searchers in the target *Pseudocercospora* species.

**Target**	**ITS**	**ACT**	**Elfa1**
P. fijiensis	GU269747.1	GU320449.1	GU384459.1
P. eumusae	GU269747.1	GU320449.1	GU384459.1
P. ulei	EF543264.1	KC800732.1	GU384459.1
P. macadamia	GU269747.1	GU320449.1	GU384459.1
