# Supplementary material for: High-quality genome assembly of Pseudocercospora ulei the main threat to natural rubber trees
Source: Genet Mol Biol. 2022 Jan 5;45(1):e50510051. doi: 10.1590/1678-4685-GMB-2021-0051 (PMC8762716; doi:10.1590/1678-4685-GMB-2021-0051)
Supplement: Table S5 - [file 1415-4757-GMB-45-1-e20210051-s5.pdf]

Genetics and Molecular Biology
https://doi.org/10.1590/1678-4685-GMB-2021-0051

Supplementary Material to "High-quality genome assembly of *Pseudocercospora ulei* the main threat to natural rubber trees"

Table S5 - Accession numbers for the genome sequences of species within Mycosphaerellaceae used for phylogenomic analyses.

Species name	accesion number	Genome Size
Cercospora berteroae	Cbert_1	33,892,839
Cercospora beticola	CB0940_V2	35,260,078
Cercospora brassicicola	ASM1336524v1	38,324,398
Cercospora canescens	C_canescens_MTCC-10836_BHU_v01	33,967,224
Cercospora cf. flagellaris	ASM535688v1	33,240,740
Cercospora cf. sigesbeckiae	ASM221750v1	34,940,764
Cercospora citrullina	ASM1336519v1	32,813,481
Cercospora kikuchii	ASM535685v1	33,223,116
Cercospora nicotianae	CNIC01	33,372,350
Cercospora sesami	ASM1336523v1	34,336,664
Cercospora sojina	ASM429982v1	40,115,407
Cercospora sojina	ASM429982v1	40,115,976
Cercospora zeina	ASM284461v1	40,755,333
Dothistroma pini	ASM211635v1	29,984,598
Dothistroma septosporum	ECU13.long	30,196,509
Exutisphaerella laricina	ASM50438v2	26,520,711
Lecanosticta acicola	ASM50434v2	28,442,312
Microcyclosporella mali	ASM278598v1	27,977,477
Mycosphaerella arachidis	ASM129726v1	33,245,410
Mycosphaerella populi	ASM215340v1	30,013,927
Mycosphaerelloides madeirae	ASM278599v1	33,743,376
Nothophaeocryptopus gaeumannii	ASM211638v1	33,964,036
Pallidocercospora crystallina	ASM366608v1	36,952,682
Passalora fulva	CfCabog12	61,103,943
Passalora sequoiae	ASM1324884v1	31,768,716
Pseudocercospora cruenta	SM1336520v1	55,623,252
Pseudocercospora eumusae	ASM157823v1	47,119,461
Pseudocercospora fijiensis	Mycfi2	74,141,167
Pseudocercospora macadamiae	ASM1297840v1	40,070,143

Species name	accesion number	Genome Size
Pseudocercospora musae	ASM157822v1	60,439,160
Pseudocercospora pini-densiflorae	ASM50436v2	43,513,371
Pseudocercospora ulei	This study	93,730,151
Ramularia coccinea	ASM415524v1	32,744,270
Ramularia collo-cygni	version 1	32,254,038
Ramularia endophylla	ASM211639v1	41,329,147
Sphaerulina musiva	Septoria musiva SO2202 v1.0	29,352,103
Sphaerulina populicola	sPop_v1	33,188,813
Zasmidium angulare	ASM278604v1	37,899,076
Zasmidium cellare	Zasce1	38,247,703
Zasmidium citrigriseum	ASM278602v1	44,999,914
Zymoseptoria ardabiliae	ASM22376v2	31,136,334
Zymoseptoria brevis	ASM96659v1	31,914,300
Zymoseptoria passerinii	ASM22382v2	28,786,283
Zymoseptoria pseudotritici	ASM22368v2	32,796,701
Zymoseptoria tritici	ASM293742v1	41,953,157
