# Supplementary material for: High-quality genome assembly of Pseudocercospora ulei the main threat to natural rubber trees
Source: Genet Mol Biol. 2022 Jan 5;45(1):e50510051. doi: 10.1590/1678-4685-GMB-2021-0051 (PMC8762716; doi:10.1590/1678-4685-GMB-2021-0051)
Supplement: Figure S2 - [file 1415-4757-GMB-45-1-e20210051-s7.pdf]

Genetics and Molecular Biology
https://doi.org/10.1590/1678-4685-GMB-2021-0051

Supplementary Material to "High-quality genome assembly of *Pseudocercospora ulei* the main threat to natural rubber trees"

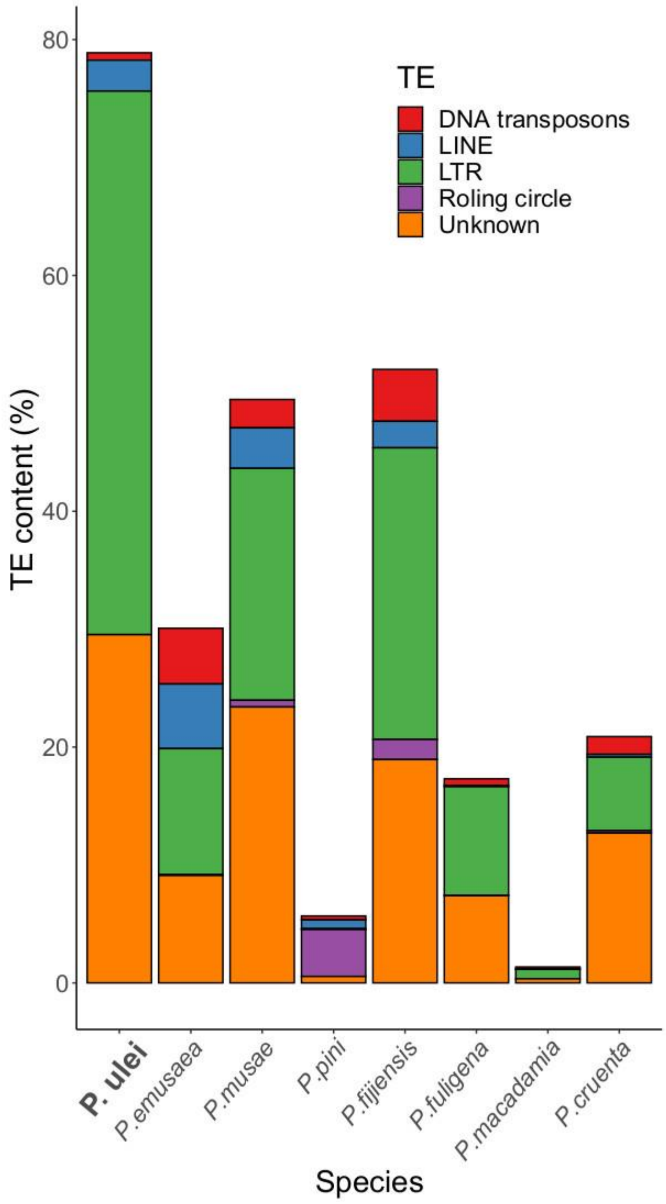

Figure S2 - Relative content of transposable elements (TEs) in the genome sequences of Pseudocercospora species. TE content: The percentage of the total genome sequence covered by TEs. DNA transposons: class II transposable elements, LINE: Long Interspersed Nuclear Elements, LTR: Long Terminal Repeats Retrotransposons, RC: Rolling-Circle transposons.
