# Supplementary material for: High-quality genome assembly of Pseudocercospora ulei the main threat to natural rubber trees
Source: Genet Mol Biol. 2022 Jan 5;45(1):e50510051. doi: 10.1590/1678-4685-GMB-2021-0051 (PMC8762716; doi:10.1590/1678-4685-GMB-2021-0051)
Supplement: Figure S3 - [file 1415-4757-GMB-45-1-e20210051-s8.pdf]

Genetics and Molecular Biology
https://doi.org/10.1590/1678-4685-GMB-2021-0051

Supplementary Material to "High-quality genome assembly of *Pseudocercospora ulei* the main threat to natural rubber trees"

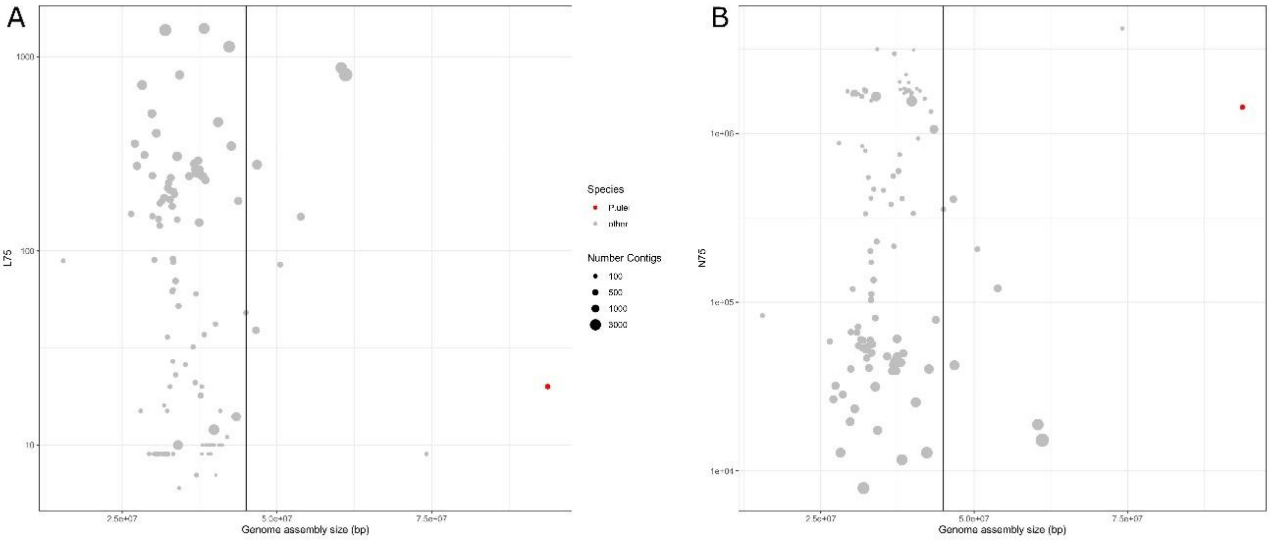

Figure S3 - Relationship between genome assembly contiguity, genome assembly size and number of contigs/scaffolds for all the genomes of species of Mycosphaerellaceae. Each genome is represented by a circle. The area of the circle is proportional to the number of contigs in the assembly. Contiguity is measured as (A) L75 (lower the better, down to the number of chromosomes) and (B) N75 (larger the better, up to the chromosome size). The figure shows 118 genome assemblies from 49 different species. The vertical line is at 45Mbp.
