# Supplementary material for: High-quality genome assembly of Pseudocercospora ulei the main threat to natural rubber trees
Source: Genet Mol Biol. 2022 Jan 5;45(1):e50510051. doi: 10.1590/1678-4685-GMB-2021-0051 (PMC8762716; doi:10.1590/1678-4685-GMB-2021-0051)
Supplement: Figure S1 - [file 1415-4757-GMB-45-1-e20210051-s6.pdf]

*Genetics and Molecular Biology*
https://doi.org/10.1590/1678-4685-GMB-2021-0051

# Supplementary Material to "High-quality genome assembly of *Pseudocercospora ulei* the main threat to natural rubber trees"

**Figure S1 -** *P. ulei* phylogenetic replacement based on genomic loci (actin, partial transcription elongation Factor 1-α (EF-1α), ITS 1 and ITS2) assessed in the *Pseudocercospora* genus.

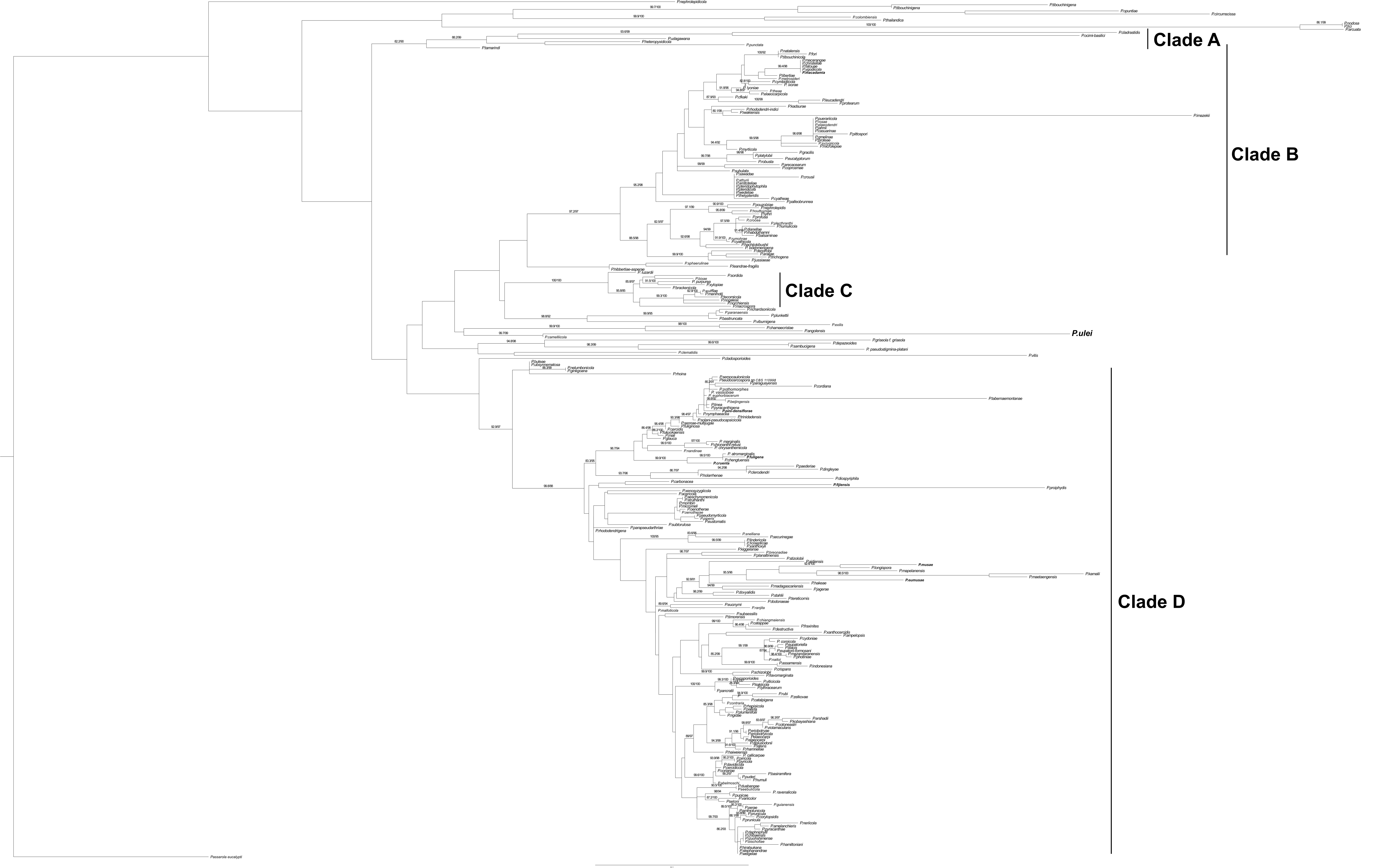
Clade A
Clade B
Clade C
P.ulei
Clade D
